# Supplementary material for: A bit or a lot on the side? Observational study of the energy content of starters, sides and desserts in major UK restaurant chains
Source: BMJ Open. 2019 Oct 7;9(10):e029679. doi: 10.1136/bmjopen-2019-029679 (PMC6797243; doi:10.1136/bmjopen-2019-029679)
Supplement: Author's manuscript [file bmjopen-2019-029679.draft_revisions.pdf]

BMJ Open

BMJ Open is committed to open peer review. As part of this commitment we make the peer review history of every article we publish publicly available.

When an article is published we post the peer reviewers' comments and the authors' responses online. We also post the versions of the paper that were used during peer review. These are the versions that the peer review comments apply to.

The versions of the paper that follow are the versions that were submitted during the peer review process. They are not the versions of record or the final published versions. They should not be cited or distributed as the published version of this manuscript.

BMJ Open is an open access journal and the full, final, typeset and author-corrected version of record of the manuscript is available on our site with no access controls, subscription charges or pay-per-view fees (http://bmjopen.bmj.com).

If you have any questions on BMJ Open's open peer review process please email info.bmjopen@bmj.com

BMJ Open

A bit or a lot on the side? An observational study of the energy content of starters, sides and desserts in major UK restaurant chains

Journal:	*BMJ Open*
Manuscript ID	bmjopen-2019-029679
Article Type:	Research
Date Submitted by the Author:	05-Feb-2019
Complete List of Authors:	Muc, Magdalena; University of Liverpool, Department of Psychological Sciences<br>Jones, Andrew; University of Liverpool, Department of Psychological Sciences<br>Roberts, Carl; Institute of Psychology, Health and Society, University of Liverpool, UK, Department of Psychological Sciences,<br>Sheen, Florence; University of Liverpool<br>Haynes, Ashleigh ; University of Liverpool<br>Robinson, Eric ; Institute of Psychology, Health and Society, University of Liverpool, UK, Department of Psychological Sciences,
Keywords:	food environment, eating out, restaurant food, kilocalories, obesity

SCHOLARONE™
Manuscripts

A bit or a lot on the side? An observational study of the energy content of starters, sides and desserts in major UK restaurant chains

Magdalena Muc[1], Andrew Jones[1], Carl Alexander Roberts[1], Florence Sheen[1], Ashleigh Haynes[1] & Eric Robinson[1]

[1] Department of Psychological Sciences, University of Liverpool, L69 7ZA, UK

Correspondence: Eric Robinson, Department of Psychological Sciences, University of Liverpool, L69 7ZA, UK. Email: eric.robinson@liv.ac.uk

Magdalena Muc, Institute of Psychology, Health & Society, University of Liverpool, L69 7ZA, UK. Email: mmuc@liverpool.ac.uk

Total word count: 3068

Key words: food environment; eating out; restaurant food; kilocalories; obesity

For peer review only

Abstract

Objectives: Main meals served by UK restaurants often have an excessive energy content, but the energy content of other menu sections has not been examined. Our objective was to examine the kilocalorie (kcal) content of starter, side and dessert dishes served in major UK restaurant chains, comparing the kcal content of these dishes in fast-food and full-service restaurants.

Design: Observational study.

Setting: Menu and nutritional information provided online by major UK restaurant chains.

Method: During October to November 2018, we accessed websites of restaurant chains with 50 or more outlets in the UK. Menu items that constituted starter, side or dessert dishes were identified and the kcal content of these dishes was extracted. Any accompanying beverage kcals were not included.

Main outcome measures: The mean kcal content of dishes and the proportion of dishes exceeding public health recommendations for energy content in a main meal (>600kcals).

Results: A total of 1009 dishes (212 starters, 317 sides and 480 desserts) from 27 restaurant chains (21 full-service, 6 fast-food) were included. The mean kcal content of eligible dishes was 477.0 (SE=25.9) for starters, 321.5 (SE=20.3) for sides and 488.4 (SE=35.05) for desserts. The percentage of dishes which exceeded 600kcals was 26.4% for starters, 21.8% for sides and 20.4% for desserts. Compared to fast-food chains, desserts offered at full service restaurants were on average more calorific and were significantly more likely to exceed 600kcal.

Conclusions: The average energy content of sides, starters and desserts sold in major UK restaurants is high. One in four starters and one in five sides and desserts in UK chain restaurants exceed the recommended energy intake for an entire meal.

For peer review only

Strengths and limitations of this study

- This is the first study of which we are aware to assess the energy content of starter, side and dessert dishes in the UK eating out sector.
- Our findings will be of use to future evaluations of how the out of home food sector respond to voluntary or mandatory public health actions through food product reformulation.
- Smaller chains and independent restaurants were not included, however there is evidence that both chain and non-chain restaurants tend to serve highly calorific foods
- We could only use the nutrition data that restaurants made available, which excluded several dishes from our analyses.

For peer review only

Introduction

Overweight and obesity are now common in most of the developed world. For example, in the UK two in three adults and one in three children are now classed as being overweight or obese [1]. Although obesity is a multifactorial disease, it is clear that changes to the food environment has been a key factor driving the global obesity epidemic [2,3]. Eating out of the home is becoming increasingly common, with 39% of adults reporting eating out at least once a week in a recent UK study [4]. Eating out of the home is associated with higher energy consumption and research suggests that frequently eating out of the home is a risk factor for obesity [5]. The consumption of 'fast-food' meals has been widely identified as a cause for concern, due to the low nutritional quality and high energy content of meals served in these restaurants [6]. Because of this, the out of home food sector has now been identified as an area for public health policy intervention in the US [7] and UK government are currently considering similar policy action [8]. However, most of the research on the nutritional quality of food eaten out of the home has been conducted in North America, a region with a particularly high prevalence of obesity [6,9,10]. There has been little research examining the nutritional quality of food sold out of the home in the UK, although a small study of meals sold in independent small scale takeaway outlets has shown that energy content can be excessive [11]. In a recent study we examined the kilocalorie content of main meals sold by major restaurant chains in the UK [12]. We found that the average kilocalorie content of main meals was high and very few meals adhered to public health recommendations for main meal kilocalorie consumption (≤600kcals) recently suggested by Public Health England [13]. Moreover, we found that main meals sold by full-service restaurants tended to be more calorific than those sold by fast-food restaurants, which is consistent with data from North American restaurants [14]. However, previous research has focused on main meal dishes and as consumers regularly order starters, sides and/or desserts as part of their meal out of the home,

For peer review only

the aims of the present study were to assess the average energy content of starter, side and dessert dishes sold in major UK restaurant chains. Based on [12] Robinson et al. (2018), we also examined how common it was for starter, side and desserts dishes to exceed the amount of calories recommended for an entire meal whether these dishes would be more calorific at full-service, as opposed to fast-food restaurants.

Methods

We pre-registered the study protocol and analysis plan on the Open Science Framework (https://osf.io/6cfdb/)

Restaurant sampling. Previously [12] we identified restaurant chains with ≥ 50 outlets in the UK by consulting market reports listing restaurants with the largest number of UK outlets, and market research ranking UK restaurant chains by annual turnover, popularity, number of users, and numbers of outlets [15-18]. If the number of UK outlets was not provided on a restaurant website, this information was requested by email.

Characterising restaurant types. As in [12] we classified restaurant chains as 'fast-food' or 'full-service' restaurants using the following definition of fast-food restaurants: *'Restaurants that primarily provide consumers with largely pre-prepared 'quick' meals with little or no table service, with in-store seating and in which take-away orders are likely to account for a significant proportion of orders'*. Therefore, coffee shops and take-away only outlets were not considered eligible. Previously two researchers independently categorised each of the included restaurant as fast-food or full-service with any disagreements resolved by a third researcher [12].

Data sources. To access current menus and nutritional information, two researchers visited the restaurants' UK web pages during October and November 2018 and accessed online versions of current menus. If a restaurant only had a downloadable menu (PDF), and no website menu, we used the former. If there were several menus (e.g. specials menus), only

the 'main menu' was used for coding. If there was no menu clearly labelled as the 'main' menu then we used the restaurant's 'evening menu'.

Starters, sides, desserts menu options. We examined the kcal content of starters, sides and dessert menu options. We defined a starter/side/dessert item as being a menu option that is not a main meal dish, is an individually sold food item and can be ordered on its own, as opposed to a more specific addition to a menu item (e.g. steak sauce, ice cream toppings). We excluded menu items that could not be ordered by all consumers (e.g. items from senior citizens menu section, children's menu section) and excluded platters and sharers (unless the menu indicated the number of people per serving) as we could not confidently identify what combination of sharing menu options would constitute a starter, side or dessert for one person. Small plates (tapas) were not eligible unless they were part of a section of the menu that was labelled as starters, side dishes or desserts. We also excluded menu items with unspecified portion size, such as "unlimited" or "bottomless" options as we would not be able to calculate energy content. In instances in which a menu option could be customized at the request of the patron for an additional charge (for example add extra toppings), we only extracted the default composition of the menu option. In instances in which a menu option required a customer to make an explicit choice (e.g. choice of topping for a starter or dessert accompaniment), we identified all possible configurations for the item and record each as an individual menu item (e.g. chocolate cake with ice cream, chocolate cake with custard) If a menu item appeared on the menu as served with a drink of choice, we excluded it as a scoping exercise indicated that this was uncommon and our focus is on energy content of food items. Finally, to minimize effects of season, we only included options that were available all year round and/or not sold everyday (e.g. only on specific days, such as 'soup of the day').

For peer review only

Two researchers (F.S and C.A.R) independently identified menu options from each restaurant and a third researcher (M.M) checked their eligibility according to the protocol and resolved any discrepancies. As there was a very high number of menu sections that were not eligible (e.g. main meals, sharers, drinks, children's menu, Sunday menu) coders did not record a classification (eligible vs. not eligible) for every item on each menu. As in (12) we used an approximated inter-coder consistency by calculating the number of menu items deemed eligible by both coders vs. the number of menu items included by only one of the coders.

Extraction of dish kcal content. As above F.S and C.A.R accessed the online nutritional information for each restaurant (October 2018) and extracted the number of kcals per menu item. M.M cross-checked kcal extraction for accuracy.

Statistical analysis

Primary analyses – average number of kcals: Menu items were nested within individual restaurants so we planned to use multi-level analyses. We first examined if a multi-level analysis was appropriate for starters, sides and dessert kcals separately by examining the portioning of variance attributed to differences in kcals between restaurants (between restaurant variance/(between restaurant variance + within restaurant variance)). We examined the multilevel model fit by comparing the loglikelihood ratio statistic (loglikelihood of the multilevel model - loglikelihood of the single-level model) to a chi-squared distribution with 1 degree of freedom. We used bootstrapping (500 samples) to improve the accuracy of parameter values and reduce bias in parameter estimates. Statistical significance ($p < .05$) indicated meaningful variation in kcals of menu items between restaurants and a multilevel model was used. In all statistical tests α was set at .05 and we report 95% confidence intervals for significance testing. Where multilevel modelling was not appropriate we used conventional frequentist statistics, maintaining $p < .05$ as the level of statistical significance.

For peer review only

Secondary analyses. Public Health England recommends that adults do not exceed 600kcals for a complete meal at lunch and dinner (8). There are no specific recommendations for individual components (e.g. energy from sides) of a meal, so we examined how common it was for starters, sides and desserts to be excessive in kcal content by calculating the proportion of menu items that exceed an entire meal's worth of kcals (600kcals). We examined differences between the two restaurant types (fast-food vs. full-service) by using one-level binary logistic regressions. Because variability in menu item kcal content between restaurant types may be in part explained by the two types of restaurant serving different types of dishes, we examined whether there were dishes that were routinely sold by both types of restaurant (e.g. side of fries/chips, salad) and we compared the average number of kcals for these dish types by restaurant type.

Results

Restaurants. Fifty-two eligible restaurant chains were identified and of these 30 restaurants had available menus and nutritional information. We requested this from the remaining chains but only one provided this information. Because we examined main meal accompaniments (starters, sides, desserts) we excluded four restaurants that only tended to sell individual food items that customers choose from to form a meal (e.g. pieces of chicken, pieces of sushi) leaving 27 restaurants in the final sample (n=6 fast-food, n=21 full-service restaurants). See Table 1 for restaurants included.

Menu items. Of all the menu items identified by either of two coders (1494), the first coder identified 74 items which were not identified by the second coder (95.1% agreement) and the second coder identified 35 items not identified by the first coder (97.7% agreement), indicating reasonable consistency between the two coders in identifying eligible dishes. The items in disagreement were then reviewed by a third researcher and after these discrepancies

For peer review only

were resolved, the final number of eligible dishes was 1361. We were able to extract kcal information for 1009 dishes (74.1% of eligible items) and the remaining dishes were treated as missing data and not included in analyses. See Table 1 for number of eligible dishes per restaurant.

Mean kcal content of menu items (Table 1). For all three groups (starters, sides and desserts) two-level model (dishes within restaurants) was a better fit of the data than a single level model. The variance partition coefficient; the total residual variance which is attributable to restaurants rather than individual dishes was 14.7% (model fit: $\chi2(1) = 18.14$, $p < .001$) for starters, 51.8% (model fit: $\chi2(1) = 123.41$, $p < .001$) for sides, and 45.1% (model fit: $\chi2(1) = 198.08$, $p < .001$) for desserts, indicating that multi-level modelling was appropriate. In a two-level model (individual dishes nested within restaurants), the average number of kcals for starters was 477.0 (SE=25.9), for sides was 321.5 (SE=20.3) and for desserts was 488.4 (SE=35.05).

Next, we compared the average number of kcal in sides and in desserts between fast-food and full-service restaurants, as there were no starters identified in the fast-food restaurants. Type of restaurant (fast-food vs. full-service) was not a significant predictor of kcal content for sides, $\beta = 3.19$, SE = 11.84 (95% CIs -20.02 to 26.40), $p = .79$) indicating that sides offered at fast-food restaurants had on average only 3.19 kcals more energy than sides from fast-food restaurants. Desserts had on average 249.47 more kcal in full-service than in fast-food chains ($\beta = 249.47$, SE = 69.74 (95% CIs 112.78 to 386.16), $p = .001$) and this difference was statistically significant.

Mean kcal content of specific dish types (Table 2). The most common side dish available was chips/fries. To compare the average kcal content of chips between the fast-food and full-service restaurants we selected only chips/fries menu options that were plain, with no sauces or spices, toppings or extras and were served as sides. That resulted in inclusion of 40 menu

items, offered in 20 restaurants (out of the 27), including five out of six fast-food chains (n=13 items), and 15 out of 21 full-service chains (n=27 items). The small number of items eligible for this sub-analysis did not lend itself to multilevel analysis so we used a t-test to compare the types of restaurants. The average number of kcals was 461.6 (SE=37.8) across all restaurants. Chips/fries in full-service restaurants had on average 226.2kcals more energy than in fast-food restaurants (535.1kcal vs 308.9kcal) and the type of restaurant (full-service vs. fast-food) was a significant predictor ($p < .01$). Ice-cream dishes were the most frequently served dessert across restaurants. We selected only ice-creams made of dairy cream, therefore items such as sorbets, vegan ice creams, and other desserts that included ice-cream (such as cake with ice cream) were excluded from the comparison. Ice-creams were served in 19 out of 27 restaurants (4 of 6 fast-foods and 15 of 21 full-service), with a total of 128 items (24 in fast foods and 104 in full-service). The average amount of kcals in ice-cream dishes was 383.4 (SE=23.7). Full-service restaurant ice-cream dishes had on average 178.3 kcals more than fast-food ice-cream dishes (416.8kcal vs. 238.5kcal) and this difference was statistically significant ($p < .01$).

Menu items >600kcals. Of the 212 starters identified, 56 (26.4%) exceeded 600kcals per dish and all starter dishes were from full-service restaurants. Of the 317 side dishes, 69 (21.8%) had >600kcal. Logistic regression models examining proportion of dishes >600kcals demonstrated significant variance at the restaurant level (Wald Test Statistic = 8.81, p = .003). The proportion of sides >600kcals was not significantly larger in sit down restaurants compared to fast food restaurants (OR = 0.98 (95% CIs: 0.14 to 6.55, p = .982). Among the 480 identified desserts, 98 (20.4%) exceeded 600kcals. Logistic regression models examining proportion of dishes >600kcals demonstrated significant variance at the restaurant level (Wald Test Statistic = 7.68, p = .006). The proportion of desserts exceeding the 600kcal

was 13.7 times larger in sit-down compared to fast food restaurants (OR = 13.69: 95% CIs 2.27 to 83.10, p = .001).

Discussion

The present study examined the energy content of starter, side and dessert dishes sold by major UK restaurant chains. We found that the average number of kilocalories in starter dishes, side dishes and dessert dishes was 477, 322 and 488 kilocalories respectively. We also examined the proportion of these dishes that we deemed to be 'excessive' by identifying those with more than 600 kilocalories; the recommended kilocalorie content of a full lunch or dinner meal in the UK [19]. We identified that one in four starters and one in five sides and desserts exceeded the amount of energy recommended for a full meal. Results also indicated that kilocalorie content of dishes was associated with restaurant type. When comparing types of restaurants, we found that desserts were significantly higher in kilocalories in full-service vs. fast-food restaurants.

Our results are in line with studies that have examined the energy content of North American restaurant food and a recent UK study [10,12,14,20,21]. Based on these results, the average energy content of a three-course meal (starter, main meal, dessert) in a major chain restaurant in the UK would be approximately 1,787 kilocalories, which equates to close to three times the recommended energy intake for a main meal, and 89% of the recommended daily consumption of kilocalories for women or 71% for men. Although individual energy requirements vary according to levels of physical activity, age, gender, and body mass, frequent eating out of home combined with the relatively high energy content of restaurant dishes (including starters, sides and desserts) may contribute to excessive energy intake that is now common in the UK and other high-income countries. The present research has

For peer review only

relevance to public health policy and our results also suggest that policy actions which result in the reduction of the energy content of restaurant food are urgently needed. In September 2018, the UK government launched an open consultation on kilocalorie labelling for food and drink served outside of the home. As our study shows, starters, sides and desserts can be highly calorific and, in some cases, exceed the amount of energy recommended for a single meal. We also found a high degree of variability in kilocalorie content in similar dishes across restaurants, which may make it difficult for consumers to estimate energy content without access to nutritional information. For example, the most calorific portion of chips/fries offered in studied restaurant chains had nearly 12 times more than the least calorific (107kcal and 1256kcal) and it was common for ice-cream desserts to vary dramatically in kilocalorie content. Therefore, mandatory kilocalorie labelling in the UK out of home food sector would be appropriate. The present study is the first of which we are aware of that assesses the energy content of starter, side and dessert dishes in the UK eating out sector, these and the results may be of use to future evaluations of how the out of home food sector respond to voluntary or mandatory public health actions through food product reformulation.

Limitations

The main limitation of our study was that we were unable to include smaller chains or independent restaurants, however the evidence from US suggest that both chain and non-chain restaurants tend to serve highly calorific foods [10]. As there are no guidelines for the limits of calories in different courses of the meal, we examined the proportion of meals exceeding Public Health England's recommendation of 600 kcals or less per entire meal (lunch or dinner). A further limitation of the study was that we were only able to make use of nutrition data from restaurants that made this information available, which excluded several

For peer review only - http://bmjopen.bmj.com/site/about/guidelines.xhtml

dishes from our analyses. This presents a potential source of bias if the kilocalorie content of restaurants that do not provide nutrition information differs to that of restaurants that do. It is also possible that restaurant-provided nutrition information is inaccurate, although research suggests that it is unlikely [22].

Conclusions

The energy content of sides, starters and desserts sold in major UK restaurants is high. One in four starters and one in five sides and desserts in UK chain restaurants exceed the recommended energy intake for an entire meal.

Data Sharing

The final data sets containing restaurant dishes' descriptions and number of kcals for each restaurant used in analyses are available online at https://osf.io/cd597/

Funding

The MRC (MR/N00218/1) part fund ER's salary. The views expressed in this publication are those of the authors and not necessarily those of the MRC.

Competing interests

All authors have completed the ICMJE uniform disclosure form at www.icmje.org/coi_disclosure.pdf. ER has been a named investigator on research projects

For peer review only

funded by the American Beverage Association but does not consider this funding a conflict of interest.

Author statement and Contribution

ER and MM designed the study. MM, CR, FS contributed to data collection. AH provided an advice and expertise at all stages and helped solving eligibility disagreements. AJ and MM were responsible for data analysis. MM was responsible for initial drafting of the paper and all authors approved the manuscript and had full access to the data.

Ethical approval

As the study involved no human or animal participants and made use of publicly available information ethical approval was not required

Transparency

MM acts as the guarantor for this work and confirms that the manuscript is an accurate, transparent and honest account of the study, that no important aspects of the study have been omitted and that any discrepancies from the study as planned have been explained.

References

1. Health matters: obesity and the food environment. 2018. https://www.gov.uk/government/publications/health-matters-obesity-and-the-food-environment/health-matters-obesity-and-the-food-environment--2.

For peer review only

2. Livingstone MBE, Pourshahidi LK. Portion Size and Obesity. *Adv Nutr* 2014; **5**(6): 829-34.

3. MacKay S. Legislative solutions to unhealthy eating and obesity in Australia. *Public Health* 2011; **125**(12): 896-904.

4. Adams J, Goffe L, Brown T, et al. Frequency and socio-demographic correlates of eating meals out and take-away meals at home: cross-sectional analysis of the UK national diet and nutrition survey, waves 1–4 (2008–12). *Int J Behav Nutr Phys Act* 2015; **12**(1): 51.

5. Bowman SA, Vinyard BT. Fast Food Consumption of U.S. Adults: Impact on Energy and Nutrient Intakes and Overweight Status: *J Am Coll Nutr*. 23 (2) (pp 163-168), 2004. Date of Publication: April 2004.; 2004.

6. Bauer KW, Hearst MO, Earnest AA, French SA, Oakes JM, Harnack LJ. Energy content of U.S. fast-food restaurant offerings: 14-year trends. *Am J Prev Med* 2012; **43**(5): 490-7.

7. Rubin R. Will Posting Nutritional Information on Menus Prod Diners to Make Healthier Choices? *JAMA* 2018; **319**(19): 1969-71.

8. Calorie labelling for food and drink served outside of the home. 2018. https://www.gov.uk/government/consultations/calorie-labelling-for-food-and-drink-served-outside-of-the-home.

9. Urban LE, Lichtenstein AH, Gary CE, et al. The energy content of restaurant foods without stated calorie information. *JAMA Intern Med* 2013; **173**(14): 1292-9.

10. Urban LE, Weber JL, Heyman MB, et al. Energy contents of frequently ordered restaurant meals and comparison with human energy requirements and US Department of Agriculture database information: a multisite randomized study. *J Acad Nutr Diet* 2016; **116**(4): 590-8. e6.

11. Jaworowska A, M. Blackham T, Long R, et al. *Nutr Food Sci* 2014; **44**(5): 414-30.

For peer review only

12. Robinson E, Jones A, Whitelock V, R. Mead B, Haynes A. (Over)eating out at major UK restaurant chains: observational study of energy content of main meals. *BMJ* 2018.

13. https://www.gov.uk/government/news/plans-to-cut-excess-calorie-consumption-unveiled.

14. Scourboutakos MJ, L'Abbe MR. Restaurant menus: calories, caloric density, and serving size. *Am j prev med* 2012; **43**(3): 249-55.

15. Mintel. Mintel Market Sizes, Restaurants in UK(2017). Report accessed via University of Liverpool Library, Liverpool, UK. March 2018.

16. Mintel. Mintel Market Sizes, Fast-food and Takeaways in UK (2016). Report accessed via University of Liverpool Library, Liverpool, UK. Accessed 1st March 2018

17. Statista. Leading restaurant chains ranked by number of users in the United Kingdom (UK) from 2017. 2017. Report accessed 1st March from https://www.statista.com/statistics/586234/restaurant-chains-usage-in-the-uk-by-number-of-users/

18. Statista. Leading restaurant chains ranked by number of users in the United Kingdom (UK) from 2017. 2017. Report accessed 1st March from https://www.statista.com/statistics/586234/restaurant-chains-usage-in-the-uk-by-number-of-users/

19. PHE. Calorie reduction: the scope and ambition for action. 2018.

20. Cawley J, Susskind A, Willage B. The Impact of Information Disclosure on Consumer Behavior: Evidence from a Randomized Field Experiment of Calorie Labels on Restaurant Menus. National Bureau of Economic Research, 2018.

21. Auchincloss AH, Leonberg BL, Glanz K, Bellitz S, Ricchezza A, Jervis A. Nutritional value of meals at full-service restaurant chains. *J Nutr Educ Behav* 2014; **46**(1): 75-81.

22. Urban LE, McCrory MA, Dallal GE, et al. Accuracy of Stated Energy Contents of Restaurant Foods. *JAMA* 2011; **306**(3): 287-93.

For peer review only

For peer review only

Table 1. Kilocalorie content of dishes from eligible restaurant chains included in analyses

			Starters		Sides		Desserts	
Restaurant type	Restaurant chain name	N	Number	Mean (SD) kcals	Number	Mean (SD) kcal per dish	Number	Mean (SD) kcals
Fast-food	Burger King	39	-	NA	19	332.1 (136.7)	20	311.5 (153.4)
	KFC	83	-	NA	73	562.9 (240.1)	10	309.5 (81.1)
	Leon	25	-	NA	10	209.3 (65.0)	15	270 (81.0)
	McDonalds	23	-	NA	6	216.3 (167.1)	17	242 (115.5)
	Subway	5	-	NA	1	705 (-)	4	213.8 (2.2)
	Wimpy	20	-	NA	12	282.2 (168.8)	8	456.3 (230.2)
			-					
	Fast-food restaurants (N=6) [b]	195	-	NA	121	453.6 (249.7)	74	297.2 (142.4)
	n (%) >600kcal	NA	-	NA	40 (33.1)		3 (4.1)	
Full-service	all bar one	11	-	NA	6	447.7 (140.0)	5	587.2 (222.6)
	Ask	89	27	565.7 (278.0)	9	315.2 (336.1)	53	273.2 (137.3)
	Bills	33	19	318.9 (123.4)	5	265.4 (161.7)	9	535.4 (298.2)
	Chef and Brewer	39	8	481.1 (124.8)	13	302.6 (213.4)	18	486.1 (300.3)
	Ember Inns	29	7	307.6 (102.9)	8	206.1 (135.2)	14	522.2 (135.1)
	Flaming Grill	26	4	644.8 (111.4)	14	459.4 (233.9)	8	767.1 (343.7)
	Harvester	34	14	424.5 (119.2)	9	254 (148.7)	11	670.9 (156.2)
	Hungry horse	44	18	660.2 (247.5)	16	454.3 (280.0)	10	867.9 (517.6)
	JD Wetherspoons	24	-	NA	14	406.1 (325.9)	10	571.3 (169.3)
	Nando's	40	6	486 (265.5)	24	365 (320.7)	10	330 (217.4)
	Old English Inn	87	11	433.5 (199.6)	10	364.4 (209.4)	66	408.3 (141.5)
	Pizza Express	49	11	379.5 (196.4)	3	328.7 (126.0)	35	467.8 (97.9)
	Pizza Hut	18	11	463.6 (107.7)	4	412.5 (158.2)	3	624.7 (82.7)
	Sizzling Pubs	36	13	477.7 (167.0)	10	391.6 (227.4)	13	723.5 (210.8)
	Slug and Lettuce	17	-	NA	7	754 (656.4)	9	400.9 (147.7)
	Stone house	33	18	622.6 (329.9)	2	88 (26.9)	13	686.4 (230.0)
	Table Table	30	9	455.9 (154.4)	12	303.3 (159.7)	9	519.3 (223.1)
	Toby's Carvery	33	10	423.3 (134.3)	-	-	23	671.5 (251.2)
	Vintage Inns	27	9	357.7 (271.9)	8	239.5 (206.7)	10	738.9 (345.9)
	Wagamama	35	-	NA	21	326 (120.1)	14	354 (115.8)
	Zizzi	81	17	575.6 (155.1)	1	222 (-)	63	246.5 (150.0)
	Full-service restaurants (N=21) [b]	815	212	488 (227.7)	196	362.9 (270.0)	406	430.5 (251.3)
	n (%) >600kcal		56 (26.4)		29 (14.8)		95 (23.4	

For peer review only

[b] For descriptive purposes, values in this row represent the mean (SD) of individual restaurant values for mean kcals per dish

- indicates absence of dish from restaurant chain menu

For peer review only

Table 2. Kilocalorie content of chips/fries and ice-cream dishes from eligible restaurant chains included in analyses

		Chips/Fries		Ice-cream
Restaurant chain	N	Mean kcal per serving (SD)	N	Mean kcal per serving (SD)
Burger King	4	342.5 (113.2)	6	168.3 (80.6)
KFC	2	372.5 (95.5)	4	253.8 (94.2)
Leon	2	174.0 (-)	-	-
McDonalds	3	339.3 (103.5)	10	211.0 (76.6)
Subway	-	-	-	-
Wimpy	2	267.5 (0.7)	4	397.3 (252.5)
Fast-food chains [b]	13	308.9 (101.7)	24	238.5 (138.6)
All bar one	1	505.0 (-)	-	-
Ask	-	-	17	268.8 (71.4)
Bills	2	429.5 (113.8)	1	107.0 (-)
Chef and Brewer	3	478.7 (133.5)	1	951.0 (-)
Ember Inns	-	-	1	338.0 (-)
Flaming Grill	1	546.0 (-)	1	1421.0 (-)
Harvester	2	469.5 (47.4)	-	-
Hungry horse	3	530 (112.9)	3	1223.7 (903)
JD Wetherspoons	1	955.0 (-)	-	-
Nando's	3	680.3 (503.8)	4	140.5 (28.5)
Old English Inn	1	764.0 (-)	12	498.5 (197.1)
Pizza Express	-	-	-	-
Pizza Hut	2	493.5 (195.9)	21	438.0 (79.8)
Sizzling Pubs	1	503.0 (-)	5	688.8 (286)
Slug and Lettuce	1	1187.0 (-)	-	-
Stone house	1	107.0 (-)	1	800.0 (-)
Table Table	3	347.3 (17.2)	3	444.3 (263.6)
Toby's Carvery	-	-	4	559 (147.2)
Vintage Inns	2	493.5 (13.4)	-	-
Wagamama	-	-	6	388.5 (104.6)
Zizzi	-	-	24	266.9 (147.4)
Full-service chains [b]	27	535.1 (254.1)	104	416.8 (289.2)

[b] For descriptive purposes, values in this row represent the mean (SD) of individual restaurant values for mean chips/fries dishes kcals and mean ice-creams dishes kcals

- indicates absence of dish from restaurant chain menu

(-) indicates absence of SD as only one eligible dish from restaurant

For peer review only

BMJ Open

A bit or a lot on the side? An observational study of the energy content of starters, sides and desserts in major UK restaurant chains

Journal:	*BMJ Open*
Manuscript ID	bmjopen-2019-029679.R1
Article Type:	Original research
Date Submitted by the Author:	07-Aug-2019

Complete List of Authors:	Muc, Magdalena; University of Liverpool, Department of Psychological Sciences<br>Jones, Andrew; University of Liverpool, Department of Psychological Sciences<br>Roberts, Carl; Institute of Psychology, Health and Society, University of Liverpool, UK, Department of Psychological Sciences,<br>Sheen, Florence; University of Liverpool<br>Haynes, Ashleigh ; University of Liverpool<br>Robinson, Eric ; Institute of Psychology, Health and Society, University of Liverpool, UK, Department of Psychological Sciences,

**Primary Subject Heading**:	Health policy
Secondary Subject Heading:	Public health
Keywords:	food environment, eating out, restaurant food, kilocalories, obesity

SCHOLARONE™ Manuscripts

A bit or a lot on the side? An observational study of the energy content of starters, sides and desserts in major UK restaurant chains

Magdalena Muc[1], Andrew Jones[1], Carl Alexander Roberts[1], Florence Sheen[1], Ashleigh Haynes[1] & Eric Robinson[1]

[1] Department of Psychological Sciences, University of Liverpool, L69 7ZA, UK

Correspondence: Eric Robinson, Department of Psychological Sciences, University of Liverpool, L69 7ZA, UK. Email: eric.robinson@liv.ac.uk

Magdalena Muc, Institute of Psychology, Health & Society, University of Liverpool, L69 7ZA, UK. Email: mmuc@liverpool.ac.uk

Total word count: 3598

Key words: food environment; eating out; restaurant food; kilocalories; obesity

For peer review only

Abstract

Objectives: Our objective was to examine the kilocalorie (kcal) content of starters, sides and desserts served in major UK restaurant chains, comparing the kcal content of these dishes in fast-food and full-service restaurants.

Design: Observational study.

Setting: Menu and nutritional information provided online by major UK restaurant chains.

Method: During October to November 2018, we accessed websites of restaurant chains with 50 or more outlets in the UK. Menu items that constituted starters, sides or desserts were identified and their kcal content was extracted. Accompanying beverages were not included. We used multilevel modelling to examine whether mean kcal content of dishes differed in fast-food vs. full-service restaurants.

Main outcome measures: The mean kcal content of dishes and the proportion of dishes exceeding public health recommendations for energy content in a main meal (>600kcals).

Results: A total of 1009 dishes (212 starters, 318 sides and 479 desserts) from 27 restaurant chains (21 full-service, 6 fast-food) were included. The mean kcal content of eligible dishes was 488.0 (SE=15.6) for starters, 397.5 (SE=14.9) for sides and 430.6 (SE=11.5) for desserts. The percentage of dishes exceeding 600kcals was 26.4% for starters, 21.7% for sides and 20.5% for desserts. Compared to fast-food chains, desserts offered at full-service restaurants were on average more calorific and were significantly more likely to exceed 600kcal.

Conclusions: The average energy content of sides, starters and desserts sold in major UK restaurants is high. One in four starters and one in five sides and desserts in UK chain restaurants exceed the recommended energy intake for an entire meal.

review only

Strengths and limitations of this study

- This is the first study of which we are aware to assess the energy content of starters, sides and desserts in the UK eating out sector.
- Our findings will be of use to future evaluations of how the out of home food sector respond to voluntary or mandatory public health actions through food product reformulation.
- Smaller chains and independent restaurants were not included, however studies indicate that chain and non-chain restaurants tend to serve highly calorific foods.
- We could only use the nutrition data that restaurants made available, which excluded several dishes from our analyses.

For peer review only

Introduction

Overweight and obesity are now common in most of the developed world. For example, in the UK two in three adults and one in three children are now classed as having overweight or obesity [1]. Although obesity is a multifactorial disease, it is clear that changes to the food environment have been a key factor driving the global obesity epidemic [2 3]. Eating out of the home is becoming increasingly common, with 39% of adults reporting eating out at least once a week in a recent UK study [4]. Eating out of the home is associated with higher energy consumption and research suggests that frequently eating out of the home is a risk factor for obesity [5]. The consumption of 'fast-food' meals has been widely identified as a cause for concern, due to the low nutritional quality and high energy content of meals served in these restaurants [6]. Because of this, the out of home food sector has now been identified as an area for public health policy intervention in the US [7] and UK government are currently considering similar policy action [8]. However, most of the research on the nutritional quality of food eaten out of the home has been conducted in North America, a region with a particularly high prevalence of obesity [6 9 10]. There has been little research examining the nutritional quality of food sold out of the home in the UK, although a small study of meals sold in independent small scale takeaway outlets has shown that energy content can be excessive [11]. In a recent study we examined the kilocalorie content of main meals sold by major restaurant chains in the UK [12]. We found that the average kilocalorie content of main meals was high and very few meals adhered to public health recommendations for main meal kilocalorie consumption (≤600kcals) recently suggested by Public Health England [13]. Moreover, we found that main meals sold by full-service restaurants tended to be more calorific than those sold by fast-food restaurants, which is consistent with data from North American restaurants [14]. However, previous research has focused on main meals and consumers eating out can be offered a choice of starters, sides and/or desserts on restaurant

For peer review only

menus. The aim of the present study was to assess the average energy content of starters, sides and desserts sold in major UK restaurant chains. Based on Robinson et al. (2018) [12], we also examined how common it was for starters, sides and desserts to exceed the amount of calories recommended for an entire meal and whether these dishes were more calorific at full-service, as opposed to fast-food restaurants.

Methods

This is an observational study of the energy content of menu items across large chain restaurants in the UK. We pre-registered the study protocol and analysis plan on the Open Science Framework (https://osf.io/6cfdb/).

Patient and Public Involvement. No patients or public were involved in this study.

Restaurant sampling. Previously [12] we identified restaurant chains with ≥ 50 outlets in the UK by consulting market reports listing restaurants with the largest number of UK outlets, and market research ranking UK restaurant chains by annual turnover, popularity, number of users, and numbers of outlets [15-18]. If the number of UK outlets was not provided on a restaurant website, this information was requested by email.

Characterising restaurant types. As in Robinson et al. (2018) [12] we classified restaurant chains as 'fast-food' or 'full-service' restaurants using the following definition of fast-food restaurants: *'Restaurants that primarily provide consumers with largely pre-prepared 'quick' meals with little or no table service, with in-store seating and in which take-away orders are likely to account for a significant proportion of orders'*. We classified full-service chains as restaurants where consumers primarily order and are served while seated at a table [19]. Therefore, coffee shops and take-away only outlets were not considered eligible. Previously two researchers independently categorised each of the included restaurant as fast-food or full-service with any disagreements resolved by a third researcher [12].

For peer review only

Data sources. To access current menus and nutritional information, two researchers visited the restaurants' UK web pages during October and November 2018 and accessed online versions of current menus. If a specific geographical location was required to access a restaurant chain menu we chose London (largest city in the UK) and the first listed location. If a restaurant only had a downloadable menu (PDF), and no website menu, we used the former. If there were several menus (e.g. specials menus), only the 'main menu' was used for coding. If there was no menu clearly labelled as the 'main' menu then we used the restaurant's 'evening menu'.

Starters, sides, desserts menu options. We examined the kcal content of starters, sides and dessert menu options. We defined a starter/side/dessert item as being a menu option that is not a main meal dish, is an individually sold food item and can be ordered on its own, as opposed to a more specific addition to a menu item (e.g. steak sauce, ice cream toppings). We excluded menu items that could not be ordered by all consumers (e.g. items from senior citizens menu section, children's menu section) and excluded platters and sharers (unless the menu indicated the number of people per serving) as we could not confidently identify what combination of sharing menu options would constitute a starter, side or dessert for one person. Small plates (tapas) were not eligible unless they were part of a section of the menu that was labelled as starters, sides or desserts. We also excluded menu items with unspecified portion size, such as "unlimited" or "bottomless" options as we would not be able to calculate energy content. In instances in which a menu option could be customized at the request of the patron for an additional charge (for example add extra toppings), we only extracted the default composition of the menu option. In instances in which a menu option required a customer to make an explicit choice (e.g. choice of topping for a starter or dessert accompaniment), we identified all possible configurations for the item and recorded each as an individual menu item (e.g. chocolate cake with ice cream, chocolate cake with custard). If

For peer review only

a menu item appeared on the menu as served with a drink of choice, we excluded it as a scoping exercise indicated that this was uncommon and our focus is on energy content of food items. Finally, to minimize effects of season, we only included options that were available all year round and sold everyday (e.g. we excluded dishes sold only on specific days, such as ‘soup of the day’).

Two researchers independently identified menu options from each restaurant and a third researcher checked their eligibility according to the protocol and resolved any discrepancies (October 2018). If menu sections were not specifically labelled as starters, sides or desserts, researchers categorised individual menu items according to the menu section they would typically be found under in UK restaurants. If there was a disagreement between two researchers a third researcher made the final decision. As there was a very high number of menu sections that were not eligible (e.g. main meals, sharers, drinks, children’s menu, Sunday menu) researchers did not record a classification (eligible vs. not eligible) for every item on each menu. As in Robinson et al (2018)[12] we used an approximated inter-coder consistency by calculating the number of menu items deemed eligible by both researchers vs. the number of menu items included by only one of the researchers.

Because variability in menu item kcal content between restaurant types may be in part explained by the two types of restaurant serving different types of dishes we examined whether there were dishes that were routinely sold by both types of restaurant (e.g. side of fries/chips, salad) and compared the average number of kcals for these dishes by restaurant type. Since the names of the same dishes could vary between menus, coding of these items was completed by one researcher and cross-checked by a second researcher.

Extraction of dish kcal content. Two researchers accessed the online nutritional information for each restaurant (November 2018) and extracted the number of kcals per menu item. A third researcher independently cross-checked kcal extraction for accuracy.

For peer review only

Statistical analysis

Primary analyses – average number of kcals: Menu items were nested within individual restaurants so we planned to use multi-level analyses (levels: menu item, restaurant) with random intercept at the restaurant level and fixed slopes. We first examined if a multi-level analysis was appropriate for starters, sides and dessert kcals separately by examining the portioning of variance attributed to differences in kcals between restaurants (between restaurant variance/ (between restaurant variance + within restaurant variance)). We examined the multilevel model fit by comparing the loglikelihood ratio statistic (loglikelihood of the multilevel model - loglikelihood of the single-level model) to a chi-squared distribution with 1 degree of freedom. We used bootstrapping (500 samples) to improve the accuracy of parameter values and reduce bias in parameter estimates. Statistical significance ($p < .05$) indicated meaningful variation in kcals of menu items between restaurants and a multilevel model was used. In all statistical tests α was set at .05 and we report 95% confidence intervals for significance testing. Where multilevel modelling was not appropriate we used conventional frequentist statistics, maintaining $p < .05$ as the level of statistical significance.

Secondary analyses. Public Health England recommends that adults do not exceed 600kcals for a complete meal at lunch and dinner [13]. There are no specific recommendations for individual components (e.g. energy from sides) of a meal, so we examined how common it was for starters, sides and desserts to be excessive in kcal content by calculating the proportion of menu items that exceed an entire meal's worth of kcals (600kcals). We examined differences between the two restaurant types (fast-food vs. full-service) by using a multi-level binary logistic regressions when appropriate.

Results

For peer review only

Restaurants. Fifty-two eligible restaurant chains were identified and of these 30 restaurants had available menus and nutritional information. We requested this from the remaining chains but only one provided this information. Because we examined main meal accompaniments (starters, sides, desserts) we excluded four restaurants that only tended to sell individual food items that customers choose from to form a meal (e.g. pieces of chicken, pieces of sushi) leaving 27 restaurants in the final sample (n=6 fast-food, n=21 full-service restaurants). See Table 1 for restaurants included.

Menu items. Of all the menu items identified by either of two coders (1494), the first coder identified 74 items which were not identified by the second coder (95.1% agreement) and the second coder identified 35 items not identified by the first coder (97.7% agreement), indicating reasonable consistency between the two coders in identifying eligible dishes. The items in disagreement were then reviewed by a third researcher and after these discrepancies were resolved, the final number of eligible dishes was 1361. We were able to extract kcal information for 1009 dishes (74.1% of eligible items) and the remaining dishes were treated as missing data and not included in analyses. The missing information for the 25.9% of the items was due to lack of nutritional information provided by restaurants. See Table 1 for number of eligible dishes per restaurant.

Mean kcal content of menu items (Table 1). For all three groups (starters, sides and desserts) two-level model (dishes within restaurants) was a better fit of the data than a single level model. The variance partition coefficient; the total residual variance which is attributable to restaurants rather than individual dishes was 14.7% (model fit: χ^2 (1) = 18.1, $p < .001$) for starters, 13.8% (model fit: χ^2 (1) = 35.0, $p < .001$) for sides, and 45.0% (model fit: χ^2 (1) = 197.5, $p < .001$) for desserts, indicating that multi-level modelling was appropriate. In a one-level model (for the descriptive purposes), the average number of kcals for starters was 488.0 (SE=15.6), for sides was 397.5 (SE=14.9) and for desserts was 430.6 (SE=11.5).

For peer review only - http://bmjopen.bmj.com/site/about/guidelines.xhtml

Next, we used a two-level model to compare the average number of kcal in sides and in desserts between fast-food and full-service restaurants, as there were no starters identified in the fast-food restaurants. Type of restaurant (fast-food vs. full-service) was not a significant predictor of kcal content for sides (β = 0.1, SE = 2.8 (95% CIs -5.5 to 5.6), p =.49) indicating that sides offered at fast-food restaurants had on average only 0.1 kcals more energy than sides from fast-food restaurants. Desserts had on average 241.2 more kcal in full-service than in fast-food chains (β = 241.2, SE = 65.4 (95% CIs 113.0 to 369.4), p = .001) and this difference was statistically significant.

Mean kcal content of specific dish types (Table 2). The most common side available was chips/fries. To compare the average kcal content of chips between the fast-food and full-service restaurants we selected only chips/fries menu options that were made of potato, plain, with no sauces or spices, toppings or extras and were served as sides. The inclusion criteria resulted in 40 eligible menu items, offered in 19 restaurants (out of the 27), including five out of six fast-food chains (n=13 items), and 14 out of 21 full-service chains (n=27 items). The small number of items eligible for this sub-analysis did not lend itself to multilevel analysis so we used Welch's t-test to compare the types of restaurants. The average number of kcals was 441.9 (SE=33.1) across all restaurants. Chips/fries in full-service restaurants had on average 197.0 kcals more than in fast-food restaurants (505.9 kcal vs 308.9kcal) and this difference was statistically significant (t (38) = 3.9, p < .01, d = 1.2). Ice-cream dishes were the most frequently served dessert across restaurants. We selected only ice-creams made of dairy cream, therefore items such as sorbets, vegan ice creams (or combinations of flavours including either of these), and other desserts that included ice-cream (such as cake with ice cream) were excluded from the comparison. Ice-creams were served in 19 out of 27 restaurants (4 of 6 fast-foods and 15 of 21 full-service), with a total of 114 items (24 in fast foods and 90 in full-service). The average amount of kcals in ice-cream dishes was 389.2

(SE=27.1). Full-service restaurant ice-cream dishes had on average 190.9 kcals more than fast-food ice-cream dishes (429.4kcal vs. 238.5kcal) and this difference was statistically significant (t (85) = 4.5, $p < .001$, $d = 0.8$).

Menu items >600kcals. Of the 212 starters identified, 56 (26.4%) exceeded 600kcals per dish and all starters were from full-service restaurants. Of the 318 sides, 69 (21.7 %) had >600kcal. Multi-level logistic regression models demonstrated the proportion of sides >600kcals was not significantly larger in fast-food restaurants compared to full service restaurants (Wald statistic (1) = 4.32, $p = .04$ OR = 1.52 (95% CIs: 0.14 to 16.10), $p =.48$). Among the 479 identified desserts, 98 (20.5 %) exceeded 600kcals. A multi-level logistic regression model demonstrated that the proportion of desserts exceeding the 600kcal was 14 times larger in full-service compared to fast food restaurants (Wald statistic (1) = 7.7, $p < .01$; OR = 14.01 (95% CIs 1.95 to 101.49), $p < .01$).

Discussion

The present study examined the energy content of starters, sides and desserts sold by major UK restaurant chains. We found that the average number of kilocalories in starters, sides and desserts was 488.0 (SE=15.6), 397.5 (SE=14.9) and 430.6 (SE=11.5) kilocalories respectively. We also examined the proportion of these dishes that we deemed to be 'excessive' by identifying those with more than 600 kilocalories; the recommended kilocalorie content of a full lunch or dinner meal in the UK [20]. We identified that one in four starters and one in five sides and desserts exceeded the amount of energy recommended for a full meal. Results also indicated that kilocalorie content of dishes was associated with restaurant type. When comparing types of restaurants, we found that desserts were significantly higher in kilocalories in full-service vs. fast-food restaurants.

Our results are in line with studies that have examined the energy content of North American restaurant food and a recent UK study finding an excessive number of kilocalories in menu items in the eating-out sector and a general trend for full-service restaurant menu items to on average be more calorific than fast-food restaurants [10 12 14 21-23]. Based on the results from our current study and the previous UK study of main meals [12], the average energy content of a three-course meal (starter, main meal, dessert, without the addition of an extra side) in a major chain restaurant in the UK would be approximately 1,896 kilocalories, which equates to over three times the recommended energy intake for a main meal, and 95% of the recommended daily consumption of kilocalories for women or 76% for men. Although individual energy requirements vary according to levels of physical activity, age, gender, and body mass, frequent eating out of home combined with the relatively high energy content of restaurant dishes (including starters, sides and desserts) may contribute to excessive energy intake that is now common in the UK and other high-income countries. The present research has relevance to public health policy and our results also suggest that policy actions which result in the reduction of the energy content of restaurant food are urgently needed. In September 2018, the UK government launched an open consultation on kilocalorie labelling for food and drink served outside of the home. As our study shows, starters, sides and desserts can be highly calorific and, in some cases, exceed the amount of energy recommended for a single meal. A recently published study performed two meta-analyses to study the effect of menu energy labelling on consumer choice and the energy content of menu items. It showed that there was a reduction in kilocalories ordered by consumers and a reduction in energy content of menu items provided by restaurants when the energy content of meals was displayed at the point-of-choice.[24] Thus, this research supports the proposition that menu labelling may benefit public health through two main channels; industry

For peer review only

reformulation and individual behaviour change. [25] Given that kilocalorie labelling is only likely to have a small effect on daily energy intake, a combination of this and other population-wide interventions will be required to improve diet and reduce obesity.

We also found a high degree of variability in kilocalorie content in similar dishes across restaurants, which may make it difficult for consumers to estimate energy content without access to nutritional information. For example, the most calorific portion of chips/fries offered in studied restaurant chains had nearly 12 times more than the least calorific (107kcal and 1256kcal) and it was common for ice-cream desserts to vary dramatically in kilocalorie content. Although due to the methodological challenges, we did not include smaller chains or independent restaurants in our study, evidence from US studies suggests that both chain and non-chain restaurants tend to serve highly calorific foods [10.] . Therefore, mandatory kilocalorie labelling in the UK out of home food sector would be appropriate. The present study is the first of which we are aware of that assesses the energy content of starters, sides and desserts in the UK eating out sector, and the results may be of use to future evaluations of how the out of home food sector respond to voluntary or mandatory public health actions through food product reformulation.

Limitations

As there are no guidelines for the limits of calories in different courses of the meal, we examined the proportion of meals exceeding Public Health England's recommendation of 600 kcals or less per entire meal (lunch or dinner). A further limitation of the study was that we were only able to make use of nutrition data from restaurants that made this information available, which excluded several dishes and restaurants from our analyses. This presents a potential source of bias if the kilocalorie content of restaurants that do not provide nutrition

For peer review only

information differs to that of restaurants that do. It is also possible that restaurant-provided nutrition information is inaccurate, although research suggests that any inaccuracy may be relatively small [26].

Conclusions

The energy content of sides, starters and desserts sold in major UK restaurants is high. One in four starters and one in five sides and desserts in UK chain restaurants exceed the recommended energy intake for an entire meal.

Data Sharing

The final data sets containing restaurant dishes' descriptions and number of kcals for each restaurant used in analyses are available online at https://osf.io/cd597/

Funding

The MRC (MR/N00218/1) part fund ER's salary. The views expressed in this publication are those of the authors and not necessarily those of the MRC.

Competing interests

All authors have completed the ICMJE uniform disclosure form at www.icmje.org/coi_disclosure.pdf. ER has been a named investigator on research projects

For peer review only

funded by the American Beverage Association but does not consider this funding a conflict of interest.

Author statement and Contribution

ER and MM designed the study. MM, CR, FS contributed to data collection. AH provided an advice and expertise at all stages and helped solving eligibility disagreements. AJ and MM were responsible for data analysis. MM was responsible for initial drafting of the paper and all authors approved the manuscript and had full access to the data.

Ethical approval

As the study involved no human or animal participants and made use of publicly available information ethical approval was not required

Transparency

MM acts as the guarantor for this work and confirms that the manuscript is an accurate, transparent and honest account of the study, that no important aspects of the study have been omitted and that any discrepancies from the study as planned have been explained.

References

1. Health matters: obesity and the food environment. 2018. https://www.gov.uk/government/publications/health-matters-obesity-and-the-food-environment/health-matters-obesity-and-the-food-environment--2.

For peer review only

2. Livingstone MBE, Pourshahidi LK. Portion Size and Obesity. *Adv Nutr* 2014; **5**(6): 829-34.

3. MacKay S. Legislative solutions to unhealthy eating and obesity in Australia. *Public Health* 2011; **125**(12): 896-904.

4. Adams J, Goffe L, Brown T, et al. Frequency and socio-demographic correlates of eating meals out and take-away meals at home: cross-sectional analysis of the UK national diet and nutrition survey, waves 1–4 (2008–12). *Int J Behav Nutr Phys Act* 2015; **12**(1): 51.

5. Bowman SA, Vinyard BT. Fast Food Consumption of U.S. Adults: Impact on Energy and Nutrient Intakes and Overweight Status: *J Am Coll Nutr*. 23 (2) (pp 163-168), 2004. Date of Publication: April 2004.; 2004.

6. Bauer KW, Hearst MO, Earnest AA, French SA, Oakes JM, Harnack LJ. Energy content of U.S. fast-food restaurant offerings: 14-year trends. *Am J Prev Med* 2012; **43**(5): 490-7.

7. Rubin R. Will Posting Nutritional Information on Menus Prod Diners to Make Healthier Choices? *JAMA* 2018; **319**(19): 1969-71.

8. Calorie labelling for food and drink served outside of the home. 2018. https://www.gov.uk/government/consultations/calorie-labelling-for-food-and-drink-served-outside-of-the-home.

9. Urban LE, Lichtenstein AH, Gary CE, et al. The energy content of restaurant foods without stated calorie information. *JAMA Intern Med* 2013; **173**(14): 1292-9.

10. Urban LE, Weber JL, Heyman MB, et al. Energy contents of frequently ordered restaurant meals and comparison with human energy requirements and US Department of Agriculture database information: a multisite randomized study. *J Acad Nutr Diet* 2016; **116**(4): 590-8. e6.

11. Jaworowska A, M. Blackham T, Long R, et al. *Nutr Food Sci* 2014; **44**(5): 414-30.

For peer review only

12. Robinson E, Jones A, Whitelock V, R. Mead B, Haynes A. (Over)eating out at major UK restaurant chains: observational study of energy content of main meals. *BMJ* 2018.

13. https://www.gov.uk/government/news/plans-to-cut-excess-calorie-consumption-unveiled.

14. Scourboutakos MJ, L'Abbe MR. Restaurant menus: calories, caloric density, and serving size. *Am j prev med* 2012; **43**(3): 249-55.

15. Mintel. Mintel Market Sizes, Restaurants in UK(2017). Report accessed via University of Liverpool Library, Liverpool, UK. March 2018.

16. Mintel. Mintel Market Sizes, Fast-food and Takeaways in UK (2016). Report accessed via University of Liverpool Library, Liverpool, UK. Accessed 1st March 2018

17. Statista. Leading restaurant chains ranked by number of users in the United Kingdom (UK) from 2017. 2017. Report accessed 1st March from https://www.statista.com/statistics/586234/restaurant-chains-usage-in-the-uk-by-number-of-users/

18. Statista. Leading restaurant chains ranked by number of users in the United Kingdom (UK) from 2017. 2017. Report accessed 1st March from https://www.statista.com/statistics/586234/restaurant-chains-usage-in-the-uk-by-number-of-users/

19. Mazidi M, State Key Laboratory of Molecular Developmental Biology IoGaDB, Chinese Academy of Sciences, Chaoyang, Beijing, China, University of the Chinese Academy of Sciences H, Beijing, China, et al. Higher densities of fast-food and full-service restaurants are not associated with obesity prevalence. *AJCN* 2019;**106**(2):603-13. doi: 10.3945/ajcn.116.151407

20. PHE. Calorie reduction: the scope and ambition for action. 2018.

For peer review only

21. Bleich SN, Wolfson JA, Jarlenski MP, Block JP. Restaurants with calories displayed on menus had lower calorie counts compared to restaurants without such labels. *Health aff.* 2015 Nov 1;**34**(11):1877-84.

22. Auchincloss AH, Leonberg BL, Glanz K, Bellitz S, Ricchezza A, Jervis A. Nutritional value of meals at full-service restaurant chains. *J Nutr Educ Behav* 2014; **46**(1): 75-81.

23. Schoffman DE, Davidson CR, Hales SB, et al. The Fast-Casual Conundrum: Fast-Casual Restaurant Entrees Are Higher in Calories than Fast Food. *J Acad Nutr Diet* 2016;**116**(10):1606-12. doi: 10.1016/j.jand.2016.03.020 [published Online First: 2016/05/18]

24. Zlatevska N, Neumann N, Dubelaar C. Mandatory Calorie Disclosure: A Comprehensive Analysis of Its Effect on Consumers and Retailers. *Journal of Retailing* 2018;Volume **94**(Issue 1):89-101. doi: https://doi.org/10.1016/j.jretai.2017.09.007

25. Roberts S, Pilard L, Chen J, et al. Efficacy of population-wide diabetes and obesity prevention programs: An overview of systematic reviews on proximal, intermediate, and distal outcomes and a meta-analysis of impact on BMI. *Obes Rev* 2019;**20**(7):947-63. doi: 10.1111/obr.12821 [published Online First: 2019/05/01]

26. Urban LE, McCrory MA, Dallal GE, et al. Accuracy of Stated Energy Contents of Restaurant Foods. *JAMA* 2011; **306**(3): 287-93.

For peer review only

Table 1. Kilocalorie content of dishes from eligible restaurant chains included in analyses

			Starters		Sides		Desserts	
Restaurant type	Restaurant chain name	N	Number	Mean (SD) kcals	Number	Mean (SD) kcal per dish	Number	Mean (SD) kcals
Fast-food	Burger King	39	-	NA	19	332.1 (136.7)	20	311.5 (153.4)
	KFC	83	-	NA	73	562.9 (240.1)	10	309.5 (81.1)
	Leon	25	-	NA	10	209.3 (65.0)	15	270.0 (81.0)
	McDonalds	23	-	NA	6	216.3 (167.1)	17	242.0 (115.5)
	Subway	5	-	NA	1	705.0 (-)	4	213.8 (2.2)
	Wimpy	20	-	NA	12	282.2 (168.8)	8	456.3 (230.2)
	All fast-food restaurants (N=6) [a]	195	-	NA	121	453.6 (249.7)	74	297.2 (142.4)
	n (%) >600kcal [b]	NA	-	NA	40 (33.1)		3 (4.1)	
Full-service	all bar one	11	-	NA	6	447.7 (140.0)	5	587.2 (222.6)
	Ask	89	27	565.7 (278.0)	9	315.2 (336.1)	53	273.2 (137.3)
	Bills	33	19	318.9 (123.4)	5	265.4 (161.7)	9	535.4 (298.2)
	Chef and Brewer	39	8	481.1 (124.8)	13	302.6 (213.4)	18	486.1 (300.3)
	Ember Inns	29	7	307.6 (102.9)	8	206.1 (135.2)	14	522.2 (135.1)
	Flaming Grill	26	4	644.8 (111.4)	14	459.4 (233.9)	8	767.1 (343.7)
	Harvester	34	14	424.5 (119.2)	9	254.0 (148.7)	11	670.9 (156.2)
	Hungry horse	44	18	660.2 (247.5)	16	454.3 (280.0)	10	867.9 (517.6)
	JD Wetherspoon	24	-	NA	14	406.1 (325.9)	10	571.3 (169.3)
	Nando's	40	6	486.0 (265.5)	24	365.0 (320.7)	10	330.0 (217.4)

For peer review only

	Old English Inn	87	11	433.5 (199.6)	10	364.4 (209.4)	66	408.3 (141.5)
	Pizza Express	49	11	379.5 (196.4)	3	328.7 (126.0)	35	467.8 (97.9)
	Pizza Hut	18	11	463.6 (107.7)	4	412.5 (158.2)	3	624.7 (82.7)
	Sizzling Pubs	36	13	477.7 (167.0)	10	391.6 (227.4)	13	723.5 (210.8)
	Slug and Lettuce	17	-	NA	7	754 (656.4)	9	400.9 (147.7)
	Stone house	33	18	622.6 (329.9)	2	88.0 (26.9)	13	686.4 (230.0)
	Table Table	30	9	455.9 (154.4)	12	303.3 (159.7)	9	519.3 (223.1)
	Toby's Carvery	33	10	423.3 (134.3)	-	-	23	671.5 (251.2)
	Vintage Inns	27	9	357.7 (271.9)	8	239.5 (206.7)	10	738.9 (345.9)
	Wagamama	35	-	NA	22	328.1 (117.6)	13	352.6 (120.4)
	Zizzi	81	17	575.6 (155.1)	1	222.0 (-)	63	246.5 (150.0)
	All full-service restaurants (N=21)	815	212	488.0 (227.7)	197	397.5 (265.3)	405	430.6 (251.5)
	n (%) >600kcal [a]		56 (26.4)		29 (14.7)		95 (23.5)	

[a] For descriptive purposes, values in this row represent the one-level mean (SD) of individual restaurant values for mean kcals per dish

[b] the values presented in these rows are the numbers of the dishes exceeding the 600 kcal and their representation among the total meals identified (n (%))

- indicates absence of dish from restaurant chain menu.

For peer review only

Table 2. Kilocalorie content of chips/fries and ice-cream dishes from eligible restaurant chains included in analyses

		Chips/Fries		Ice-cream
Restaurant chain	N	Mean kcal per serving (SD)	N	Mean kcal per serving (SD)
Burger King	4	342.5 (113.2)	6	168.3 (80.6)
KFC	2	372.5 (95.5)	4	253.8 (94.2)
Leon	2	174.0 (-)	-	-
McDonalds	3	339.3 (103.5)	10	211.0 (76.6)
Subway	-	-	-	-
Wimpy	2	267.5 (0.7)	4	397.3 (252.5)
All fast-food restaurants [a]	13	308.9 (101.7)	24	238.5 (138.6)
All bar one	2	452.0 (75.0)	-	-
Ask	-	-	16	272.8 (72.0)
Bills	2	429.5 (113.8)	1	107.0 (-)
Chef and Brewer	3	478.7 (133.5)	1	951.0 (-)
Ember Inns	-	-	1	338.0 (-)
Flaming Grill	1	546.0 (-)	1	1421.0 (-)
Harvester	2	469.5 (47.4)	-	-
Hungry horse	3	530.0 (112.9)	3	1223.7 (903)
JD Wetherspoon	1	955.0 (-)	-	-
Nando’s	3	680.3 (503.8)	4	140.5 (28.5)
Old English Inn	1	764.0 (-)	12	498.5 (197.1)
Pizza Express	-	-	11	481.1 (63.4)
Pizza Hut	2	493.5 (195.9)	-	-
Sizzling Pubs	1	503.0 (-)	5	688.8 (286)

For peer review only

Slug and Lettuce	-	-	-	-
Stone house	1	107.0 (-)	1	800.0 (-)
Table Table	3	347.3 (17.2)	2	596.5 (9.2)
Toby's Carvery	-	-	4	559.0 (147.2)
Vintage Inns	2	493.5 (13.4)	-	-
Wagamama	-	-	5	412.0 (97.7)
Zizzi	-	-	23	270.5 (146.7)
All Full-service restaurants [a]	27	505.9 (219.2)	90	429.4 (289.4)

[a] For descriptive purposes, values in this row represent the one-level mean (SD) of individual restaurant values for mean chips/fries dishes kcals and mean ice-creams dishes kcals

- indicates absence of dish from restaurant chain menu

(-) indicates absence of SD as only one eligible dish from restaurant

For peer review only

STROBE Statement—Checklist of items that should be included in reports of ***cross-sectional studies***

	Item No	Recommendation
**Title and abstract**	1	(*a*) Indicate the study's design with a commonly used term in the title or the abstract **1, 26**
		(*b*) Provide in the abstract an informative and balanced summary of what was done and what was found **23-43**
**Introduction**		
Background/rationale	2	Explain the scientific background and rationale for the investigation being reported **56-80**
Objectives	3	State specific objectives, including any prespecified hypotheses **80-84**
**Methods**		
Study design	4	Present key elements of study design early in the paper **86-87**
Setting	5	Describe the setting, locations, and relevant dates, including periods of recruitment, exposure, follow-up, and data collection **89-92**
Participants	6	(*a*) Give the eligibility criteria, and the sources and methods of selection of participants **NA**
Variables	7	Clearly define all outcomes, exposures, predictors, potential confounders, and effect modifiers. Give diagnostic criteria, if applicable **88 (pre-registered protocol), 94-151**
Data sources/ measurement	8*	For each variable of interest, give sources of data and details of methods of assessment (measurement). Describe comparability of assessment methods if there is more than one group **94-151**
Bias	9	Describe any efforts to address potential sources of bias **100-105, 132-142, 149-151**
Study size	10	Explain how the study size was arrived at **184-192**
Quantitative variables	11	Explain how quantitative variables were handled in the analyses. If applicable, describe which groupings were chosen and why **194-238**
Statistical methods	12 **152-174**	(*a*) Describe all statistical methods, including those used to control for confounding
		(*b*) Describe any methods used to examine subgroups and interactions
		(*c*) Explain how missing data were addressed
		(*d*) If applicable, describe analytical methods taking account of sampling strategy
		(*e*) Describe any sensitivity analyses
**Results**		
Participants	13* **177-193**	(a) Report numbers of individuals at each stage of study—eg numbers potentially eligible, examined for eligibility, confirmed eligible, included in the study, completing follow-up, and analysed
		(b) Give reasons for non-participation at each stage
		(c) Consider use of a flow diagram
Descriptive data	14*	(a) Give characteristics of study participants (eg demographic, clinical, social) and information on exposures and potential confounders **Table 1**
		(b) Indicate number of participants with missing data for each variable of interest **NA**
Outcome data	15*	Report numbers of outcome events or summary measures **194,210,230**

For peer review only - http://bmjopen.bmj.com/site/about/guidelines.xhtml

Main results	16	(*a*) Give unadjusted estimates and, if applicable, confounder-adjusted estimates and their precision (eg, 95% confidence interval). Make clear which confounders were adjusted for and why they were included **194-209**
		(*b*) Report category boundaries when continuous variables were categorized **168-172**
		(*c*) If relevant, consider translating estimates of relative risk into absolute risk for a meaningful time period **NA**
Other analyses	17	Report other analyses done—eg analyses of subgroups and interactions, and sensitivity analyses **210-238**
**Discussion**		
Key results	18	Summarise key results with reference to study objectives **240-250**
Limitations	19	Discuss limitations of the study, taking into account sources of potential bias or imprecision. Discuss both direction and magnitude of any potential bias **294-302**
Interpretation	20	Give a cautious overall interpretation of results considering objectives, limitations, multiplicity of analyses, results from similar studies, and other relevant evidence **252-292**
Generalisability	21	Discuss the generalisability (external validity) of the study results **260-278**
**Other information**		
Funding	22	Give the source of funding and the role of the funders for the present study and, if applicable, for the original study on which the present article is based **314-315**

*Give information separately for exposed and unexposed groups.

Note: An Explanation and Elaboration article discusses each checklist item and gives methodological background and published examples of transparent reporting. The STROBE checklist is best used in conjunction with this article (freely available on the Web sites of PLoS Medicine at http://www.plosmedicine.org/, Annals of Internal Medicine at http://www.annals.org/, and Epidemiology at http://www.epidem.com/). Information on the STROBE Initiative is available at www.strobe-statement.org.

For peer review only

For peer review only - http://bmjopen.bmj.com/site/about/guidelines.xhtml
